# Hepatitis B-related hepatocellular carcinoma in a 36-year-old pregnant woman: prognosis and management dilemma

**DOI:** 10.11604/pamj.2020.36.298.22658

**Published:** 2020-08-18

**Authors:** Bruce Shinga Wembulua, Kalilou Diallo, Mame Aïsse Thioubou, Jean Didier Bosenge Nguma, Noel Magloire Manga

**Affiliations:** 1 Department of Infectious and Tropical Diseases, Fann University Hospital, Dakar, Senegal,; 2 Unit of Infectious and Tropical Diseases, Assane Seck University, Hospital de la Paix, Ziguinchor, Senegal,; 3 Unit of Gastroenterology and Hepatology, Hospital de la Paix, Ziguinchor, Senegal,; 4 Department of Obstetrics and Gynecology, Kisangani University Hospital, Democratic Republic of the Congo

**Keywords:** Chronic hepatitis B infection, hepatocellular carcinoma, pregnancy

## Abstract

Management of chronic hepatitis B infection complicated by hepatocellular carcinoma (HCC) in pregnancy poses a treatment dilemma as the pregnancy accelerates disease progression and narrows the diagnostic tools and therapeutic choices. Studies have reported higher maternal and fetal losses. We share our experience with a 36-year-old pregnant woman who presented at 35 weeks' gestation with a large painful nodular liver and significant weight loss. She tested HBsAg-positive and had both clinical and laboratory features of severe liver decompensation. The abdominal ultrasound rightly described HCC on a cirrhotic background. The fetus was delivered by cesarean section but the mother died soon after.

## Introduction

More than 80% of hepatocellular carcinoma (HCC) cases in the world are attributable to hepatitis B virus (HBV) and hepatitis C virus (HCV) [1]. Although the global incidence of HCC in women is 5.5/10,000 [2], HCC during pregnancy is so rare that less than 50 such cases have been reported worldwide [3,4]. Many authors have reported more aggressive behavior of HCC during pregnancy due to elevated levels of estrogen and altered immune activity [4]. The overall management is complex as one must consider both the mother and the fetus from the diagnostic processes to treatment. This article describes our experience with a case of hepatocellular carcinoma (HCC) in pregnancy with underlying chronic hepatitis B infection and severe liver decompensation.

## Patient and observation

A 27-year-old pregnant woman presented to our facility at 35 weeks´ gestation with a 3-months history of right upper abdominal pain associated with generalized itch, tea colored urine, mastic -colored stool, and significant weight loss. No headache, blurred vision or spontaneous bleeding were mentioned. She was not a known diabetic or hypertensive and has no history of blood transfusion. Clinical signs on examination included mild pallor and cholestatic jaundice. Her blood pressure was 100/70 mmHg with a pulse rate of 98 beats/min, respiratory rate about 16/min and oral temperature of 37°C. Respiratory, cardiovascular and neurological examinations were unremarkable. The abdomen was grossly distended with visible collateral veins. Liver was enlarged, hard, nodular with a painful irregular edge. The spleen was not palpable. There was bipedal edema up to the mid shin. Fetal heart rate was 140 bpm and regular. Laboratory examination showed leukocytosis, anemia and normal platelet counts. Aspartate aminotransferase (AST) were raised (4times upper limit), total protein and albumin were low, 43g/L and 25g/L respectively, INR 1.20 and glycaemia 0.70g/L. Her alphafeto-protein (AFP) was higher (2232 ng/mL). HBsAg was positive. She tested negative to human immunodeficiency viruses (HIV) and HCV. Abdominal ultrasound showed a heterogeneous coarse liver with multiple hypoechoic lesions. Pelvic scan revealed a normal fetus with a gestational age of about 35 weeks. The diagnosis of hepatocellular carcinoma on a decompensated cirrhosis was made. She was managed with analgesia, spironolactone and tenofovir added for prevention of mother-to-child transmission of the hepatitis B infection. The plan was to have the mother´s health improved to securely induce the labour. Unfortunately, at day two of hospitalization, the patient experienced progressive loss of conscious along with epistaxis and multi-organ failure. Laboratory assessment revealed HB-8.4g/dL, WBC 37500 cells/dL, Platelets 436000/ul, INR-2.57 and recurrent hypoglycemia. Cesarean section was urgently performed and the outcome was a healthy newborn weighted 2.9kg but the mother died soon after.

## Discussion

Hepatocellular carcinoma (HCC) is rare during pregnancy because of the lower overall incidence of HCC in women of childbearing [2-4]. The exposure to higher amounts of human chorionic gonadotropin (HCG), estrogen and placental lactogen secreted from the placenta are believed to alter liver metabolism and stimulate the rapid growth of liver cancer during pregnancy [3-5]. Right upper quadrant pain, weight loss, hepatomegaly and abnormal liver enzymes found in our patient have been the most reported presentations [3,4]. Chronic HBV infection is the major cause of liver cirrhosis and HCC in sub-Saharan Africa [2]. In their review of HCC during pregnancy, Choi *et al*. [3]. Reported 64.7% case of positive HBsAg. More than 34.4% patients had liver cirrhosis. Our patient was tested HBsAg-positive and had features of liver decompensation and the ultrasound rightly described HCC on a cirrhotic background. Pregnancy presents an obstacle for diagnosing and treating HCC. Computed tomography (CT) and magnetic resonance imaging (MRI) contrast agents are not recommended because of the risk associated with exposing the fetus to radiation and other fetal problems such as post implantation fetal loss and growth retardation. Abdominal ultrasound is probably the best alternative [3,6]. Alpha-fetoprotein (AFP) developed for HCC screening, is normally detected during pregnancy secondary to placental spillover from the fetus into the maternal circulation [3,7]. Values above 600ng/mL are suggestive of HCC only in chronic HBV surface antigen (HBsAg) carriers [3]. In our observation, the diagnosis was confirmed with abdominal ultrasound and markedly elevated alpha fetoprotein (2,232ng/mL). Liver resection is the best therapeutic modality for HCC if possible [3,7]. In case of advanced-stage such as ours, termination of pregnancy as soon as possible is recommended [3]. Except cases of fibrolamellar HCC [8], primary liver cancer during pregnancy has a poor outcome [3-7]. The overall 6-month, 1-, and 2-year survival rates in the patients reported in the review of Choi *et al*. [3] were 50%, 29.5%, 18.2%, respectively. Delivery was by cesarean section in 44.82% and live births were present in only 58.7% cases. In our case, the fetus was safely delivered by cesarean section but the mother died soon after.

## Conclusion

Outcomes of HCC during pregnancy have not been good. Pregnant women should be systematically screened for HBV infection. Malignancies should be carefully approached and considered as differential diagnoses in chronic HBV surface antigen (HBsAg) carriers.
